# Effects of acupuncture for the treatment of endometriosis-related pain: A systematic review and meta-analysis

**DOI:** 10.1371/journal.pone.0186616

**Published:** 2017-10-27

**Authors:** Yang Xu, Wenli Zhao, Te Li, Ye Zhao, Huaien Bu, Shilin Song

**Affiliations:** 1 Department of Gynecology and Obstetrics, Nankai Hospital, Tianjin Academy of Integrative Medicine, Tianjin, China; 2 Graduate School, Tianjin University of Traditional Chinese Medicine, Tianjin, China; 3 Department of Neurology, Nankai Hospital, Tianjin Academy of Integrative Medicine, Tianjin, China; 4 Department of Chinese Medicine, Tianjin Hearing Impairment Specialist Hospital, Tianjin, China; 5 Department of Chemical Engineering, University of Florida, Gainesville, Florida, United States of America; 6 Department of Public Health, School of Traditional Chinese Medicine, Tianjin University of Traditional Chinese Medicine, Tianjin, China; 7 Laboratory of Anatomy, School of Integrative Medicine, Tianjin University of Traditional Chinese Medicine, Tianjin, China; Stanford University School of Medicine, UNITED STATES

## Abstract

**Background:**

Endometriosis is a multifactorial, oestrogen-dependent, inflammatory, gynaecological condition that can result in long-lasting visceral pelvic pain and infertility. Acupuncture could be an effective treatment for endometriosis and may relieve pain. Our aim in the present study was to determine the effectiveness of acupuncture as a treatment for endometriosis-related pain.

**Methods:**

In December 2016, six databases were searched for randomised controlled trials that determined the effectiveness of acupuncture in the treatment of endometriosis-related pain. Ultimately, 10 studies involving 589 patients were included. The main outcomes assessed were variation in pain level, variation in peripheral blood CA-125 level, and clinical effective rate. All analyses were performed using comprehensive meta-analysis statistical software.

**Results:**

Of the 10 studies included, only one pilot study used a placebo control and assessed blinding; the rest used various controls (medications and herbs), which were impossible to blind. The sample sizes were small in all studies, ranging from 8 to 36 patients per arm. The mean difference (MD) in pain reduction (pre- minus post-interventional pain level—measured on a 0–10-point scale) between the acupuncture and control groups was 1.36 (95% confidence intervals [CI] = 1.01–1.72, P<0.0001). Acupuncture had a positive effect on peripheral blood CA-125 levels, as compared with the control groups (MD = 5.9, 95% CI = 1.56–10.25, P = 0.008). Similarly, the effect of acupuncture on clinical effective rate was positive, as compared with the control groups (odds ratio = 2.07; 95% CI = 1.24–3.44, P = 0.005).

**Conclusions:**

Few randomised, blinded clinical trials have addressed the efficacy of acupuncture in treating endometriosis-related pain. Nonetheless, the current literature suggests that acupuncture reduces pain and serum CA-125 levels, regardless of the control intervention used. To confirm these findings, additional, blinded studies with proper controls and adequate sample sizes are needed.

## Introduction

Endometriosis is a chronic, oestrogen-dependent, inflammatory disease that affects 5%–15% of reproductive-age women, causing infertility and pain—specifically chronic pelvic pain, deep dyspareunia, dysmenorrhoea, dyschezia, and dysuria [1–4]. It may be that endometriosis-related changes are caused by plastic changes in the peripheral and central nervous systems, and such changes may in fact predispose for other long-lasting pain conditions [5]. Therefore, it is important that researchers develop strategies to alleviate pain.

Current pain therapies often involve various pharmacological and surgical treatments, and the symptoms of endometriosis are frequently treated using oestrogen–gestagen combinations or gonadotropin-releasing hormone (GnRH) agonists that block the menstrual cycle. However, many such interventions do not sufficiently affect perceived pain, and pain relapses are possible [6–8]. Furthermore, they can have considerable side effects, such as menopausal disorders, that represent an additional handicap for affected women [9].

Thus, acupuncture may serve as a complement or alternative to these treatments. The pain-alleviating effects of acupuncture have been attributed to various physiological and psychological processes, such as activation of endogenous descending pain inhibitory systems, deactivation of brain areas that transmit pain-related signals, interaction between nociceptive impulses and somato-visceral reflexes, and the expectation of symptom relief [10–12].

Cancer antigen 125 (CA-125), a well-established marker of epithelial cell ovarian cancer, is derived from coelomic epithelia, including those of the endometrium, fallopian tubes, ovaries, and peritoneum [13]. In endometriosis, CA-125 levels are elevated through stimulation of coelomic epithelia [14]. In previous studies, we have found a strong association between preoperatively elevated CA-125 levels and advanced stage of disease [15]. Similarly, Amaral et al. reported that women with more advanced degrees of endometriosis showed higher CA-125 levels in both serum and peritoneal fluid [16]. However, many studies have reported that acupuncture can reduce the level of serum CA-125, relieving the pelvic cavity pain that is associated with endometriosis [17–20]

The purpose of this systematic review and meta-analysis was to determine the effectiveness of acupuncture in treating endometriosis-related pain.

## Materials and methods

### Electronic searches

We adopted the Cochrane Menstrual Disorders and Subfertility Group (MDSG) search strategy. Reports that described (or might have described) randomised controlled trials of acupuncture in the treatment of endometriosis were obtained using the following strategy:

1. The MDSG specialised register of controlled trials was searched for any trials with endometriosis in the title, abstract, or keyword sections.
2. The following electronic databases were searched (from inception to December 2016): Cochrane Central Register of Controlled Trials (CENTRAL; The Cochrane Library), PubMed, and EMBASE.
3. Four electronic Chinese databases were examined (from inception to December 2016): the Chinese Science and Technology Journal Full-text Database (CNKI), Wanfang Data, the Chinese Biomedical Literature Database (VIP), and the China Biology Medicine (CBM) disc.

A detailed search strategy is given in S1 Appendix.

#### Searching other resources

We searched the bibliographies of the retrieved studies, narrative reviews, and meta-analyses to identify further relevant articles. Moreover, we contacted the authors to ask for the raw data. An additional search of conference abstracts was carried out on the ISI Web of Knowledge.

### Selection criteria and exclusion criteria

#### Research type

We included randomised controlled trials (RCTs).

#### Research subjects

We recruited women of reproductive age who had a laparoscopically confirmed diagnosis of endometriosis. The exclusion criteria for individual participants were primary dysmenorrhoea (in the absence of an identifiable pathological condition) and asymptomatic endometriosis.

#### Interventions

Intervention groups comprised patients who had received acupuncture therapy; control groups had received sham acupuncture, Western medicine, or Traditional Chinese Medicine.

### Outcomes

#### Variations in main pain level

Our primary treatment outcome measurement was any change in the level of pelvic pain not associated with menses or sexual activity. This outcome was assessed after 8 weeks of treatment and was based on the pain intensity question in the Endometriosis Symptom Severity Scale [21]. Using a numerical analogue scale, patients were asked to rate, from 0 to 10, pain severity during the previous 4 weeks that was not associated with menses or sexual activity. The Endometriosis Symptom Severity Scale has been validated and is sensitive to changes in endometriosis-associated pelvic pain in adults (not adolescents) enrolled in clinical trials [21].

#### Variation in peripheral blood CA-125 levels

Blood CA-125 levels were determined before and after treatment using an enzyme-linked immunosorbent assay.

#### Clinical effective rate

The overall effectiveness of acupuncture therapy was assessed subjectively and in a dichotomous manner; it was defined as the proportion of participants who experienced relief of their endometriosis-associated pain after acupuncture treatment, as indicated by the patients’ responses to the evaluation criteria. Therapeutic effects were assessed in terms of the diagnosis and treatment standards for Combined Traditional Chinese and Western medicine in the treatment of endometriosis (1991) [22]. Patients were classified in one of four groups: (1) cured—the symptoms of dysmenorrhoea, abdominal discomfort, abdominal pain, periodic rectal irritation, etc., as well as the pelvic mass, had disappeared; (2) markedly effective—abdominal pain was obviously relieved, other symptoms had improved, and the pelvic mass had narrowed by more than 50%; (3) effective—abdominal pain was relieved, other symptoms had improved, the pelvic mass had narrowed more than 33%, and dysmenorrhoea had not increased in severity three menstrual cycles after treatment; (4) failed—abdominal pain and other symptoms had not changed. The clinical effective rate was calculated using the following equation: (number of cured + markedly effective + effective cases)/ (total number of cases).

### Data extraction and quality assessment

Searches were conducted and the data were extracted by two independent researchers. Each trial identified in the search was evaluated in terms of design, eligibility criteria for participants, and outcome measures. When the researchers disagreed regarding the eligibility of a trial, they consulted a third researcher to resolve the situation. We created a form for data extraction which included (1) basic information about each trial, including the topic, first author, dateline, and journal; (2) basic information about the patients, including the number of patients in each group and their mean age; (3) the study design and intervention; and (4) the outcomes.

The quality of the trials included in this study was assessed by two other researchers in accordance with the Cochrane Handbook for Systematic Reviews of Interventions (Version 5.1.0) [23].

### Statistical analyses

All analyses were performed using comprehensive meta-analysis statistical software (RevMan 5.1.0; Cochrane Collaboration, Copenhagen, Denmark) [23]. Continuous outcome variables were analysed using a standardised measure; dichotomous variables were compared and the results were presented as odds ratios (ORs). To obtain the standard deviation (SD) of the change from baseline in the experimental intervention groups, we used the following equation, in which R_1_ = 0.5 [23]:

$$SD\left(C\right)=\sqrt{SD{\left(B\right)}^{2}+SD{\left(F\right)}^{2}-(2\times \text{R}1\times SD\left(B\right)\times SD\left(F\right)}$$

The term “*SD(B)*” represents the standard deviation before intervention, while “*SD(F)*” denotes the standard deviation after intervention.

We evaluated homogeneity among the trials using I^2^ statistics. Specifically, if I^2^ was ≥ 50%, the trials were considered heterogeneous, and a random-effects model based on a Mantel–Haenszel (MH) or inverse variance (IV) statistical approach was selected. If I^2^ was < 50%, the studies were considered homogeneous, and a fixed-effects model based on an MH or IV statistical approach was used. Pooled summary statistics of the differences in ratio or mean of the individual studies were developed. Pooled differences in ratios or means, as well as two-sided P-values, were calculated and used as criteria for determining the level of statistical significance. P-values < 0.05 were considered statistically significant. Moreover, a sensitivity analysis was conducted based on the “leave-one-out” cross-validation procedure [24].

## Results

### Study selection

**Fig 1:**
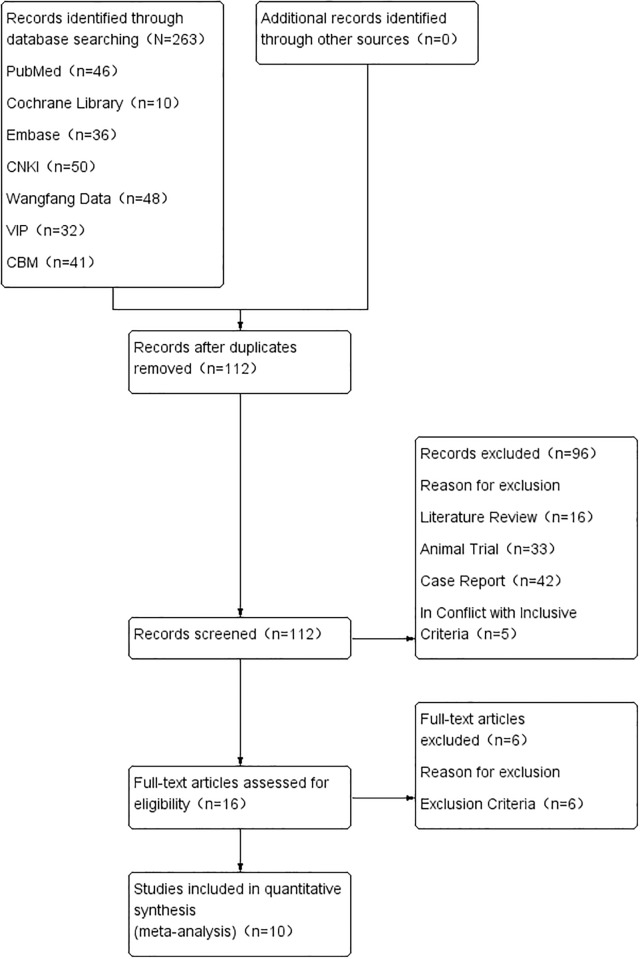
Flow chart of literature retrieval and trial selection.

A flow chart of the included and excluded studies is shown in Fig 1. Database searches yielded 46 studies from PubMed, 10 from the Cochrane Central Register of Clinical Trials, 36 from Embase, 50 from CNKI, 48 from Wanfang Data, 32 from VIP, and 41 from CBM. After removal of duplicate records, 112 studies remained. Following the first review, which was based on the title, 16 studies remained, the abstracts of which were reviewed using pre-defined eligibility criteria. A total of 16 studies were then selected for full text review and data processing. During this phase, six studies were excluded. Ultimately, 10 studies, comprising 589 participants, were included in the final meta-analysis.

### Characteristics of the included studies

#### Information regarding the included studies

The included studies comprised a total of 295 patients whose endometriosis-related pain was treated using acupuncture. A further 294 patients comprised the control group; the details of their treatment are given below. In the 10 RCTs included, the patients’ age at enrolment ranged from 13 to 52 years. The interventions were all acupuncture, while the control interventions were placebo [25] (also called sham acupuncture), Western medicine [17, 19–20, 26], and Traditional Chinese Medicine [18, 27–30] (Table 1). One RCT was conducted in Boston, while the others were from China. Other details of this kind are summarised in Table 2.

**Table 1:** Characteristics of the 10 trials identified in the literature search.

Studies	Random-isation method	Sample Size Inter-vention/Control	Age(T/C)	Outcomes	Course of Treatment	Follow-up Visit	Jadad Score
Sun YZ et al.(2006) [17]	Random number table	30/30	23–49/27–52	Clinical efficacy+symptom score+CA-125+Side effects	3 months	6 months	3
Peter M et al.(2008) [25]	Random number table	10/8	13–22	Pain score+HRQOL+IL-6 +TNF-α	8 weeks	6 months	4
Chen M et al. (2010) [27]	Random number table	34/36	23–45/22–43	Clinical efficacy+pain score	3 months	6 months	3
Xiang DF et al. (2011) [18]	Random number table	30/28	23–45/27–44	pain score+CA-125	3 months	NA	4
Wu JX et al. (2013) [26]	Random number table	30/30	23–46/26–45	Clinical efficacy+pain score	3 months	NA	3
Chen GX et al.(2014) [19]	Random number table	28/28	25–45	Pain score+SF-36+CA-125	3 months	6 months	3
Zhang XX et al. (2015) [20]	Random number table	36/36	23–45/25–46	Clinical efficacy+pain score+CA-125	6 months	12 months	3
Chen QX (2015) [28]	Random number table	30/30	22–45	Clinical efficacy+pain score+symptom score	2 months	3 months	4
Gao CY et al. (2015) [29]	Random number table	32/33	25–44/25–43	Clinical efficacy+pain score+symptom score	3 months	NA	3
Tian LY et al. (2016) [30]	Random number table	35/35	20–45	Clinical efficacy	4 months	NA	3

**Table 2:** Interventions of the 10 trials identified in the literature search.

Studies	Intervention		Treatment schedule
	Intervention group	Control group	
Sun YZ et al.(2006) [17]	Acupuncture at BL18, BL20, L23, LR14, LR13, GB25	Danazol	Treatment started 7 days before the onset of menstruation and was delivered once per day for 14 days.
Peter M et al.(2008) [25]	Acupuncture at *Jing-well* points	Sham-acupuncture	Twice per week for 8 consecutive weeks
Chen M et al. (2010) [27]	Acupuncture at CV3, CV4, extra point *Zigong*	Traditional Chinese Medicine (oral administration of *Jiawei Mojie* tablet)	Treatment started 7 days before the onset of menstruation and was delivered once per day for 14 days.
Xiang DF et al. (2011) [18]	Acupuncture at CV12, CV10, CV6, CV4, CV3	Traditional Chinese Medicine(*Tianqi Tongjing* Capsule)	Treatment started 7 days before the onset of menstruation and was delivered once every two days for 7 days
Wu JX et al. (2013) [26]	Acupuncture at CV3, CV4, extra point *Zigong*, SP6, SP8, SP10, LIV3	Mifepristone	Treatment started 7 days before the onset of menstruation and was delivered once per day for 14 days.
Chen GX et al.(2014) [19]	Acupuncture at SP6, BL23, BL32, SP10, extra point *Zigong*, CV4	Goserelin acetate	Treatment started 7 days before the onset of menstruation and was delivered 3 times per week
Zhang XX et al. (2015) [20]	Electroacupuncture at CV6, CV4, CV3, extra point *Zigong*, SP8, SP6, LI4, LIV3	Mifepristone	After *qi* arrival, G6805-I pulse electronic apparatus was attached to EX-CA1, CV4, and CV3 (continuous wave, frequency: 70 Hz, intensity: 3mA). EA was delivered once every 2 days.
Chen QX (2015) [28]	Acupuncture at CV3, CV10, CV6, CV4, CV12	Traditional Chinese Medicine	Treatment started on the first day after menstrual onset and was delivered once every 3 days.
Gao CY et al. (2015) [29]	Acupuncture at LU7, SP4, SP6, CV4, BL32, EX-B8	Traditional Chinese Medicine (oral dysmenorrhea particles)	Treatment started 7 days before the onset of menstruation and was delivered once per day for 14 days.
Tian LY et al. (2016) [30]	Acupuncture at CV6, CV4, CV3, SP6, ST36, SP10, GV20	Traditional Chinese Medicine (Modified *Gexia Zhuyu* decoction)	Treatment started 7 days before the onset of menstruation and was delivered once per day for 14 days.

#### Quality assessment

**Fig 2:**
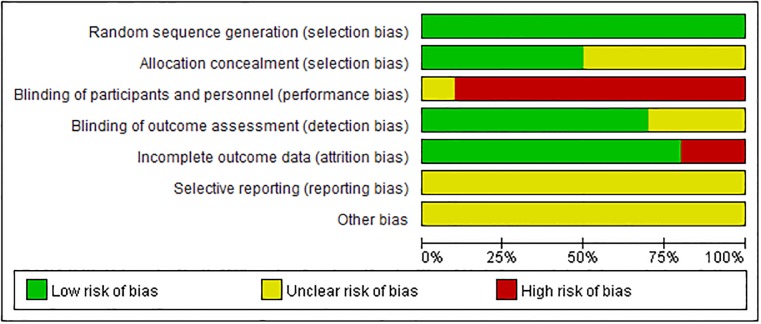
Risk of bias graph: Review authors’ judgements about each risk of bias item, presented as a percentage, across all included studies.

**Fig 3:**
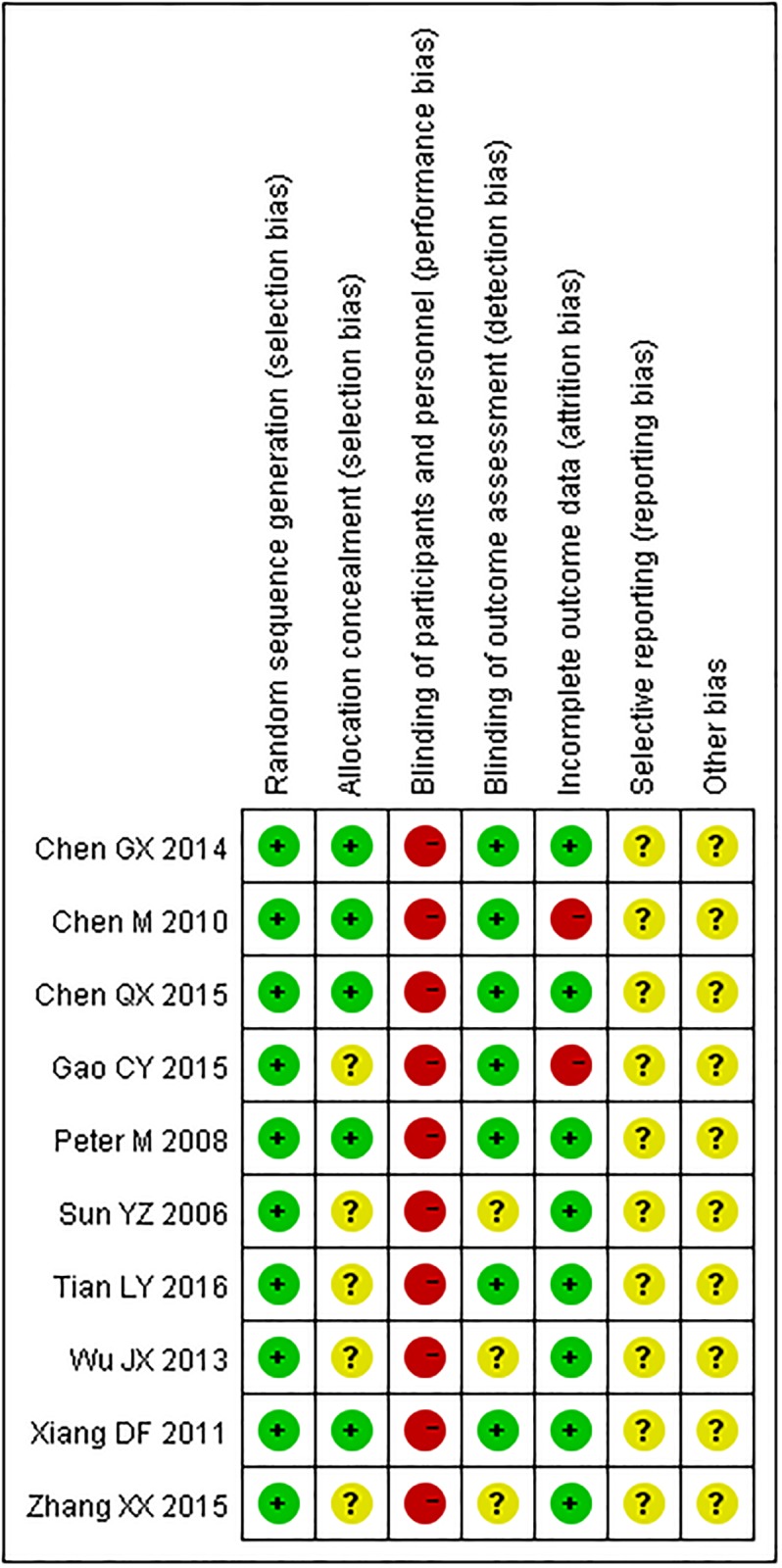
Risk of bias summary: review authors’ judgements about each risk of bias item in each included study.

The quality assessment is summarised in Figs 2 and 3.

### Clinical outcomes

#### Variation in main pain level

**Fig 4:**
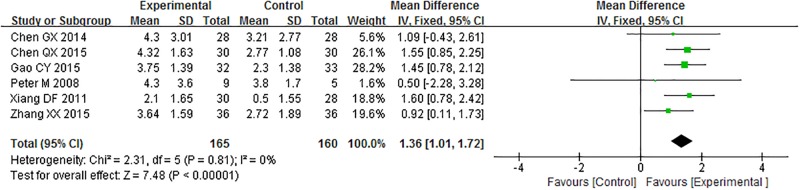
Meta-analysis of the variation in main pain level.

Six trials compared the variation in main pain level between acupuncture and control groups. Analysis of the pooled data using a fixed-effects model showed that acupuncture had a positive effect on the primary pain level, as compared with the control groups (MD = 1.36, 95% CI = 1.01–1.72, P < 0.0001; Fig 4).

#### Variation in peripheral blood CA-125 levels

**Fig 5:**
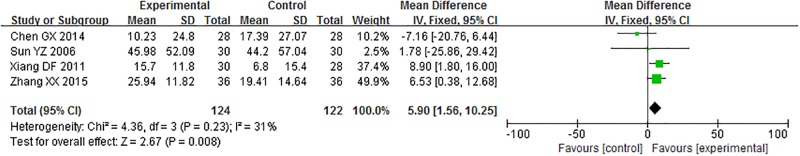
Meta-analysis of the variation in peripheral blood CA-125 levels.

Four trials compared the variation in peripheral blood CA-125 levels between acupuncture and control groups. Analysis of the pooled data using a fixed-effects model showed that acupuncture had a positive effect on the peripheral blood CA-125 level, as compared with the control groups (MD = 5.9, 95% CI = 1.56–10.25, P = 0.008; Fig 5).

#### Clinical effective rate

**Fig 6:**
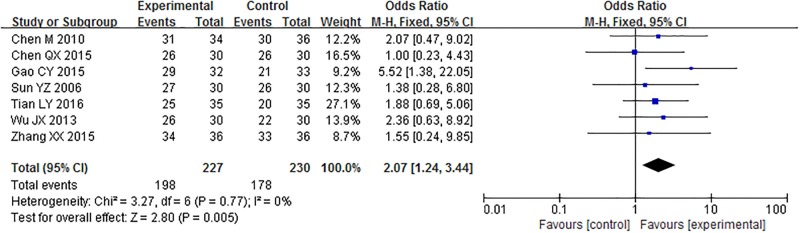
Meta-analysis of the clinical effective rate.

Seven trials compared the clinical effective rate of treatment between acupuncture and control groups. Analysis of the pooled data using a fixed-effect model showed that acupuncture had a positive effect on the clinical effective rate, as compared with the control groups (OR = 2.07, 95% CI = 1.24–3.44, P = 0.005; Fig 6).

#### Funnel plot of publication bias

**Fig 7:**
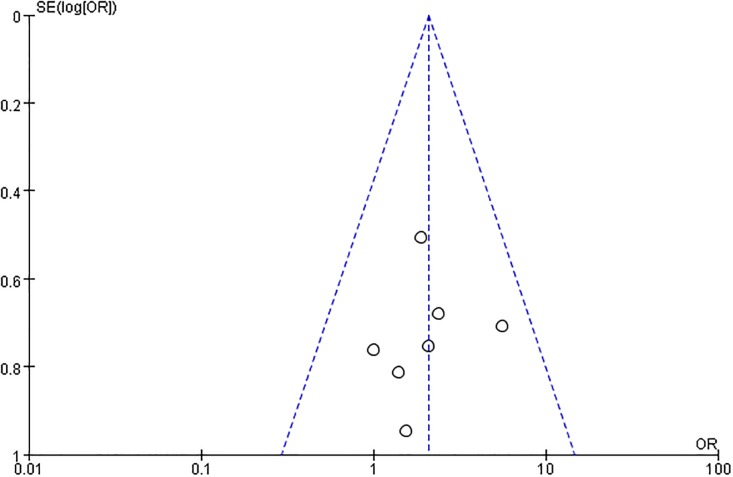
Clinical effects in the intervention groups.

Using a funnel plot, the research team analysed publication bias in all included studies (Fig 7). The outcome suggested that there was little publication bias.

### Characteristics of the excluded studies

Six studies failed to meet our inclusion criteria for the following results: (1) in one RCT comparing acupuncture with drug therapy (danazol), the pain conditions were not solely related to endometriosis [31]; (2) one study involved surgical intervention rather than acupuncture [32]; (3) one study evaluated the effectiveness of moxibustion alone and point injection, rather than moxibustion as an adjunctive therapy to acupuncture [33]; (4) four studies compared different methods of acupuncture and lacked a placebo or biomedical group [34–36].

## Discussion

### Meta-analysis of clinical effect

In the current study, we systematically reviewed the results of 10 RCTs comparing the outcomes of acupuncture with those of other therapies (sham acupuncture, Western medicine, or Traditional Chinese Medicine) in the treatment of endometriosis-related pain. Among the 10 RCTs included, six reported variations in main pain level, four reported variations in peripheral blood CA-125 levels, and seven reported the clinical effective rate of acupuncture as a treatment for endometriosis-related pain. In all 10 of the studies, the interventions were acupuncture, and the control interventions were placebo [25], Western medicine [17, 19–20, 26], or Traditional Chinese Medicine. Because so few studies were included, we did not carry out a subgroup analysis.

Only one of the included RCTs [25] selected sham acupuncture as the control intervention; fourteen participants completed this study in accordance with the protocol. Participants in the active acupuncture group (n = 9) experienced a mean 4.8-point (SD = 2.4-point) reduction on an 11-point scale (62%) in pain after 4 weeks; this differed significantly from the control group’s (n = 5) mean reduction of 1.4 points (SD = 2.1 points; P = 0.004). Reduction in pain in the acupuncture group persisted throughout the 6-month assessment. Preliminary estimates indicate that Japanese-style acupuncture may be an effective, safe, and well-tolerated adjunct therapy for endometriosis-related pelvic pain in adolescents.

Four of the included RCTs selected Western medicine as the control intervention [17, 19–20, 26]. Specifically, the studies used danazol, mifepristone, or goserelin acetate, and showed that acupuncture was better than Western medicine in relieving pain and reducing the concentration of CA-125.

Five of the RCTs selected Traditional Chinese Medicine [17, 19–20, 26] as the control intervention, and all showed that acupuncture is better than Traditional Chinese medicine in relieving pain. However, we must acknowledge that few properly blinded trials [25] have addressed this issue, and that the effects of expectation or other non-specific factors may have contributed to the benefits seen in the present study. Nonetheless, based on a single placebo-controlled study and on other studies comparing acupuncture to Western medicine and Eastern herbs, acupuncture appears to be effective in reducing pain and serum CA-125 levels in endometriosis.

Our findings were similar to those of Lund I [37] and Zhu X [38], although we considered a greater number of databases than these previous studies; we also identified more RCTs that included three outcome measures (variation in main pain level, variation in peripheral blood CA-125 level, and clinical effective rate) in our meta-analysis.

### Mechanisms of acupuncture in the treatment of endometriosis-related pain

#### Acupuncture and analgesia

Acupuncture seems to alleviate pain by increasing pain thresholds in human subjects, and it would appear to activate analgesic brain mechanisms through the release of neurohumoral factors, including adenosine, γ-aminobutyric acid, opioid peptide, acetylcholine, nitric oxide, noradrenaline, dopamine) and others [39]. Specifically, electroacupuncture can lead to the production of dopamine in the adrenal medulla [40].

#### Acupuncture and endocrine function

Endometriosis is a multifactorial, oestrogen-dependent, inflammatory, gynaecological condition. Growth of the endometrial tissue depends on oestrogen. In the human body, 90% of endogenous oestrogen is produced by the granular cells and membrane cells of the ovary, which produce the hormone in response to stimulation by follicle-stimulating hormone and luteinising hormone. In addition, oestrogen may be compounded by androstenedione from the adrenal glands [41]. Relatedly, several studies have shown that acupuncture can suppress serum oestradiol levels [42]. Thus, it may inhibit the growth of the ectopic endometrium and relieve pain.

#### Acupuncture and immune function

Evidence suggests that endometriosis has a strong immune component [43–44]. Acupuncture enhances the ability of the immune system to more actively eliminate malignant cells by increasing the ability of NK cells to kill cancer cells [45]. More specifically, acupuncture stimulation increases the cytotoxicity of NK cells by promoting cross-talk between the neurotransmitter network and the immune system; this cross-talk is mediated by nitric oxide, β-endorphins, and cytokines [46], and it is anchored by opioid and NK cell receptors.

Thus, the effect of acupuncture in the treatment of endometriosis-related pain is likely mediated by endocrine and cytokine changes, as well as by anti-inflammatory and analgesic effects.

### Limitations

The limitations of this evaluation system were as follows: (1) few of the studies discussed how the sample size was estimated, and most involved small sample sizes leading to a low precision; (2) some of the studies did not adequately report allocation concealment; failure to fully implement allocation concealment may exaggerate any curative effect observed; (3) because so few studies were included, we did not carry out a subgroup analysis; (4) three of the 10 studies [19, 28, 29] included in the final analysis were doctoral theses published in university archives. It is important that a separate analysis be performed without these thesis dissertations, which were not published in any peer-reviewed journal. In this regard, we did run the analysis again, and the conclusions did not change. (5) The results were heterogeneous because subjective indicators were used to evaluate curative effects (pain level, clinical effective rate); (6) implementation of the blinding method is important, but the included studies did not describe the implementation of the blinding method; and (7) the study was limited to Chinese- and English-language research articles, which may have introduced selection bias. Nonetheless, the overall bias was not large, so the conclusion is reliable.

### Perspectives

In future, we recommend additional, well-designed clinical trials that compare specific types of acupuncture to placebo in the treatment of endometriosis. We emphasise the importance of proper blinding and randomisation when considering other, Western treatments in clinical trials involving acupuncture.

## Conclusions

Acupuncture can alleviate the pain of dysmenorrhoea and reduce peripheral blood CA-125. As a result, the therapy could be applied as a complementary treatment for endometriosis-related pain. However, few randomised, blinded clinical trials have addressed the efficacy of acupuncture in treating endometriosis-related pain. Nonetheless, the current literature consistently finds that acupuncture yields better reductions in pain and serum CA-125 levels than do control treatments, regardless of the control intervention used. To confirm this finding, additional studies with proper controls, blinding methods, and adequate sample sizes are needed.

## Supporting information

S1 Checklist PRISMA 2009 checklist. (DOCX)

S1 Appendix Search strategy. (DOCX)
