# Supplementary material for: Effects of acupuncture for the treatment of endometriosis-related pain: A systematic review and meta-analysis
Source: PLoS One. 2017 Oct 27;12(10):e0186616. doi: 10.1371/journal.pone.0186616 (PMC5659600; doi:10.1371/journal.pone.0186616)
Supplement: S1 Appendix — (DOCX) [file pone.0186616.s002.docx]

**Appendix**

**Search strategy**

**Cochrane Central Register of Controlled Trials**

1 exp Endometriosis/

2 adenomyosis.tw.

3 Endometriosis.tw.

4 pelvic pain.tw.

5 dyspareunia.tw.

6 dyschezia.tw.

7 (pain$ adj1 defecat$).tw.

8 (pain$ adj1 intercourse).tw.

9 or/1-8

10 exp Acupuncture/

11 exp acupuncture therapy/ or exp acupressure/ or exp acupuncture analgesia/ or exp acupuncture, ear/ or exp electroacupuncture/

or exp meridians/ or exp moxibustion/

12 acupressure$.tw.

13 Acupuncture.tw.

14 (electroacupuncture or electro-acupuncture).tw.

15 meridian$.tw.

16 mox$.tw.

17 (shiatsu or tui na).tw.

18 needling.tw.

19 shu.tw.

20 acup$ point$.tw.

21 or/10-20

22 21 and 9

23 from 22 keep 1-11

**EMBASE**

1 exp Endometriosis/

2 adenomyosis.tw.

3 Endometriosis.tw.

4 pelvic pain.tw.

5 dyspareunia.tw.

6 dyschezia.tw.

7 (pain$ adj1 defecat$).tw.

8 (pain$ adj1 intercourse).tw.

9 or/1-8

10 exp Acupuncture/

11 exp acupuncture therapy/ or exp acupressure/ or exp acupuncture analgesia/ or exp acupuncture, ear/ or exp electroacupuncture/

or exp meridians/ or exp moxibustion/

12 acupressure$.tw.

13 Acupuncture.tw.

14 (electroacupuncture or electro-acupuncture).tw.

15 meridian$.tw.

16 mox$.tw.

17 (shiatsu or tui na).tw.

18 needling.tw.

19 shu.tw.

20 acup$ point$.tw.

21 or/10-20

22 21 and 9

23 Clinical Trial/

24 Randomized Controlled Trial/

25 exp randomisation/

26 Single Blind Procedure/

27 Double Blind Procedure/

28 Crossover Procedure/

29 Placebo/

30 Randomi?ed controlled trial$.tw.

31 Rct.tw.

32 random allocation.tw.

33 randomly allocated.tw.

34 allocated randomly.tw.

35 (allocated adj2 random).tw.

36 Single blind$.tw.

37 Double blind$.tw.

38 ((treble or triple) adj blind$).tw.

39 placebo$.tw.

40 prospective study/

41 or/23-40

42 case study/

43 case report.tw.

44 abstract report/ or letter/

45 or/42-44

46 41 not 45

47 22 and 46

48 limit 47 to yr=“ - 2016”

49 from 48 keep 1-18

**Ovid MEDLINE**

1 exp Endometriosis/

2 adenomyosis.tw.

3 Endometriosis.tw.

4 pelvic pain.tw.

5 dyspareunia.tw.

6 dyschezia.tw.

7 (pain$ adj1 defecat$).tw.

8 (pain$ adj1 intercourse).tw.

9 or/1-8

10 exp Acupuncture/

11 exp acupuncture therapy/ or exp acupressure/ or exp acupuncture analgesia/ or exp acupuncture, ear/ or exp electroacupuncture/

or exp meridians/ or exp moxibustion/

12 acupressure$.tw.

13 Acupuncture.tw.

14 (electroacupuncture or electro-acupuncture).tw.

15 meridian$.tw.

16 mox$.tw.

17 (shiatsu or tui na).tw.

18 needling.tw.

19 shu.tw.

20 acup$ point$.tw.

21 or/10-20

22 21 and 9

23 randomised controlled trial.pt.

24 controlled clinical trial.pt.

25 (randomised or randomised).ab.

26 placebo.ab.

27 drug therapy.fs.

28 randomly.ab.

29 trial.ab.

30 groups.ab.

31 or/23-30

32 (animals not (humans and animals)).sh.

33 31 not 32

34 33 and 22

35 from 34 keep 1-18

**China Knowledge Infrastructure (CNKI) and Traditional Chinese Medicine Database System (TCMDS). The following terms in Pingyin were used:**

1. Zi Gong Nei Mu Yi Wei Zheng (endometriosis)

2. Tong Jing (period pain)

3. Ji Fa Xing Tong Jing (secondary dysmenorrhoea)

4. 1 or 2 or 3

5. Zheng Jiu (acupuncture and moxibustion)

6. Ti Zheng (body acupuncture)

7. Er Zheng (auricular acupuncture)

8. Tou Zheng (scalp acupuncture)

9. Dian Zheng (electro-acupuncture)

10. Lin Chuang (clinical)

11. Lin Chuang Yun Yong (clinical application)

12. Lin Chuang Zhi Liao (clinical treatment)

13. Lin Chuang Yan Jiu (clinical research)

14. Lin Chuang Guan Cha (clinical observation)

15. Lin Chuang Dui Zhao (clinical comparison)

16. 5 or 6 or 7 or 9 or 10 or 11 or 12 or 13 or 14 or 15 or 16.

17. 4 and 16.
